# Untargeted Metabolomics Analysis by UHPLC-MS/MS of Soybean Plant in a Compatible Response to *Phakopsora pachyrhizi* Infection

**DOI:** 10.3390/metabo11030179

**Published:** 2021-03-19

**Authors:** Evandro Silva, José Perez da Graça, Carla Porto, Rodolpho Martin do Prado, Estela Nunes, Francismar Corrêa Marcelino-Guimarães, Mauricio Conrado Meyer, Eduardo Jorge Pilau

**Affiliations:** 1 Laboratory of Biomolecules and Mass Spectrometry, Department of Chemistry, State University of Maringá, 5790, Colombo Av, Maringá 87020-080, PR, Brazil; evandroas20@gmail.com (E.S.); cporto.silva@gmail.com (C.P.); pradorodolpho@gmail.com (R.M.d.P.); 2 Brazilian Agricultural Research Corporation Soybean, Carlos João Strass Rd, Londrina 86001-970, PR, Brazil; perezparr@gmail.com (J.P.d.G.); francismar.marcelino@embrapa.br (F.C.M.-G.); mauricio.meyer@embrapa.br (M.C.M.); 3 MsBioscience, Quintino Bocaiúva 298, Street, Maringá 87020-160, PR, Brazil; 4 Brazilian Agricultural Research Corporation Swine & Poultry, BR-153, Km 110 Rd, Concórdia 89715-899, SC, Brazil; estela.nunes@embrapa.br

**Keywords:** Asian soybean rust, soybean, metabolomics, UHPLC-MS/MS, chemometrics, GNPS, *Phakopsora pachyrhizi*

## Abstract

*Phakopsora pachyrhizi* is a biotrophic fungus, causer of the disease Asian Soybean Rust, a severe crop disease of soybean and one that demands greater investment from producers. Thus, research efforts to control this disease are still needed. We investigated the expression of metabolites in soybean plants presenting a resistant genotype inoculated with *P. pachyrhizi* through the untargeted metabolomics approach. The analysis was performed in control and inoculated plants with *P. pachyrhizi* using UHPLC-MS/MS. Principal component analysis (PCA) and the partial least squares discriminant analysis (PLS-DA), was applied to the data analysis. PCA and PLS-DA resulted in a clear separation and classification of groups between control and inoculated plants. The metabolites were putative classified and identified using the Global Natural Products Social Molecular Networking platform in flavonoids, isoflavonoids, lipids, fatty acyls, terpenes, and carboxylic acids. Flavonoids and isoflavonoids were up-regulation, while terpenes were down-regulated in response to the soybean–*P. pachyrhizi* interaction. Our data provide insights into the potential role of some metabolites as flavonoids and isoflavonoids in the plant resistance to ASR. This information could result in the development of resistant genotypes of soybean to *P. pachyrhizi*, and effective and specific products against the pathogen.

## 1. Introduction

Soybean (Glycine max (L.) Merrill) is one of the most important economic and oil crops worldwide. The major significances of soybean are related to their great nutritional value both to humans and livestock and are used to produce renewable fuels [1,2]. However, soybean crop yield is largely affected by several diseases. Asian soybean rust (ASR) disease alone was estimated to cause 10% yield losses in the north-central United States and 50% or greater in the south-eastern United States [3]. Losses can be as high as 90% when climatic conditions favor pathogens reproduction [4]. The ASR is caused by the *Phakopsora pachyrhizi* pathogen, a biotrophic fungus that can establish itself at any stage of plant development [5]. Measures to control fungal growth are centered on the use of fungicides. However, there is a growing concern about the use of such fungicides due to environmental impact, human health, and the appearance of resistant *P. pachyrhizi* strains [6]. The constant use of triazole and strobilurin fungicides, for example, has resulted in the development of increased tolerance in *P. pachyrhizi* populations [7].

Another way to fight the disease is through the use of resistant cultivars. Seven dominant resistance to *Phakopsora pachyrhizi* (*Rpp1–Rpp7*) loci, have been identified mapped at different loci for conferring immunity (without visible symptoms) or incomplete resistance which is more common than immunity. Incomplete resistance is characterized by the development of reddish-brown lesions (RB) with reduced sporulation to specific pathogen isolates [8,9,10]. However, the *Rpp* genes confer resistance to only a limited set of specific *P. pachyrhizi* isolates, and these single gene sources are not durable when used in the field, due to pathogen variability [11].

Plants have mechanisms to recognize pathogens via pathogen-associated molecular patterns (PAMPs) and prevent their development using various defensive mechanisms [12]. However, pathogens can bypass these mechanisms and propagate through the plant [13]. Plant defensive options can be the modulation of primary and secondary metabolism [14]. For example, stressed plants will produce hormones, such as salicylic acid (SA), jasmonic acid (JA), and ethylene (ET), which activate plant defense genes causing changes in metabolic pathways that produce a great diversity of secondary metabolites defense [15,16].

Untargeted metabolomics is an analytical approach employed to identify as many metabolites as possible in a given sample, which can be used to understand plant-pathogen interactions [17]. Plant specimens are complex matrices composed of a myriad of metabolites. Thus, analytical methods based on mass spectrometry (MS) should be encouraged to identify and quantify such metabolites [18]. Moreover, the MS technique provides high sensitivity, resolution, detection, and precision [19]. The coupling of liquid chromatography to mass spectrometry is used to further resolve the complexity of samples via a prior separation, thus improving the detection of the metabolites. This ensures relatively high sensitivity, repeatability, and selectivity [20].

One of the drawbacks of using ultra-high-performance liquid chromatography coupled to high-resolution mass spectrometry (UHPLC-MS/MS) is the massive amount of data generated. Tools to assist data organization, processing, and metabolite annotation are often used to assist and reduce time in the data mining stage. Of those, chemometric strategies are widely explored to extract relevant information from a data set. Multivariate analysis methods, such as principal component analysis (PCA) and the partial least squares discriminant analysis (PLS-DA), where the molecular features that contribute most to the variation or separation are identified for further analysis, are the most used in the field of metabolomics [21]. Both methods describe a set of variables measured in a set of individuals in a more condensed form, this is obtained by projecting the data in a reduced space so that it represents as much of the original information as possible [22].

For the metabolite annotation, selected by the chemometric analysis, emerging approaches to computational analysis based on MS should be employed, such as Molecular Networking based on fragmentation mass spectra (MS/MS). The MS/MS spectra are compared with reference spectra on the Global Social Natural Products Molecular Networking (GNPS) platform to annotate molecules and discover putative analogs [23]. We previously reported the metabolomics of a soybean genotype susceptible to *P. pachyrhizi* using mass spectrometry combined with the Molecular Networking tool. The results revealed a significant production of secondary defense metabolites in the plant infected by *P. pachyrhizi*, which originated from the phenylpropanoid and flavonoid pathways [24]. In this study, we performed an untargeted metabolomics analysis of a soybean plant containing the *Rpp3* gene [25], that shows incomplete resistance with RB-type lesion, in response to infection by *P. pachyrhizi*. In this case, liquid chromatography coupled to mass spectrometry (UHPLC-MS/MS), integrated with multivariate analysis and Molecular Networking were employed to elucidate changes in metabolic profiles related to plant defense responses. The results can identify metabolic pathways involved in the systemic induced resistance against infection by *P. pachyrhizi*, in plants of *Rpp3* gene, providing subsidies for the unveiling of defense mechanisms of the plant, and the production of new lines of resistant soybean.

## 2. Results

### 2.1. Visual Observation of Symptoms

First, the changes in metabolism in the soybean plant when they were inoculated or not by *P. pachyrhizi* after 14 days post-inoculation were evaluated. This period was sufficient for the pathogen to infect the leaves of the plant (spots and color changes), therefore, comparisons of the metabolome between the groups of control and inoculated plants could be evaluated. The initial symptoms of the disease are seen on the back of the leaf and are characterized by spots darker than the leaf tissue (Supplementary Figure S1) compared to the leaf tissue of a healthy plant, with a corresponding protuberance (Supplementary Figure S2), lesion known as uredinia [26]. The spores of the fungus are produced in the uredinia, which can be seen five to eight days after infection. Plant genotypes resistant to ASR show RB lesions, with reduced levels of sporulation of uredias in the lesions. (Supplementary Figure S1), while susceptible genotypes have tan-colored lesions (TAN) and are characterized by the increased formation of uredias.

### 2.2. UHPLC-ESI(+)-MS/MS Analysis

The UHPLC-ESI(+)-MS/MS technique was employed to evaluate the metabolite profile of soybean plants inoculated or not with *P. pachyrhizi* spores and to obtain the largest number of significant metabolites in the plant-pathogen interaction. Chromatograms were obtained in triplicate from UHPLC-ESI-(+)-MS/MS analysis for each plant, totaling 18 chromatograms for control plants and 18 chromatograms for inoculated plants (Supplementary Figure S3).

The MS chromatograms indicated different metabolic profiles in comparison with inoculated and non-inoculated plants. Base peak chromatograms (BPC) showed differences in chromatographic peaks (absence/presence and intensities) between the retention time interval 5.0–12.5 min (Supplementary Figure S3). However, only the visual inspection of BPC is not enough to discriminate changes in plant metabolism that occurred after infection by the pathogen. For this reason, chemometric analyses were used in the UHPLC-ESI(+)-MS/MS data.

### 2.3. Data Processing and Chemometric Analysis

**Figure 1:**
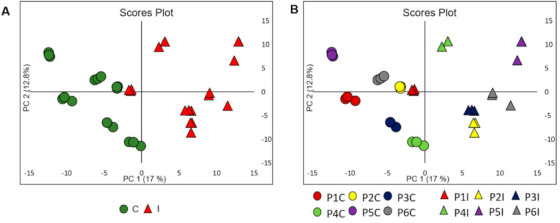
Unsupervised chemometric modeling (UHPLC-ESI-(+)-MS/MS data): (**A**) PCA scores of 18 samples (triplicate of 6 control plants and 6 inoculated plants), green circles correspond to control plant samples (C), red triangle corresponds to inoculated plant samples (I). (**B**) The scores plot in (**A**) but colored according to triplicate each plant (P). 5 PCs explained 51.6% of the total data variance.

The data matrix of molecular features (rt, *m/z*) was obtained after pre-processing the data and exported to the MetaboAnalyst 3.0 online software for the use of multivariate analyses. The first analysis was performed by using PCA, an unsupervised method usually employed to determine patterns between multivariate samples. The PCA analysis showed a clear tendency of separation between the data set of samples from control plants and inoculated with the pathogen (Figure 1A), thus reflecting differential metabolic characteristics in these two groups. It was also possible to observe that the PCA analysis was reproductive for each plant (Figure 1B). The first main component (PC1) explained 17.0% of the total variability of the data set while the second main component (PC2) explained 12.8% of the total variability of the data set.

For a better understanding of the metabolic characteristics and interpretation of the results obtained by the unsupervised analysis model, the PLS-DA method was applied. The method consists of highlighting specific similarities or differences between the samples, preferably organizing the main components that have relationships between important variables and can be specific to the group of interest. Besides, statistics such as variable importance in the projection (VIP) obtained by the PLS-DA method and can be used to select the most important variables, [27] allowing the identification of characteristics of the metabolites responsible for the discrimination between classes or groups.

**Figure 2:**
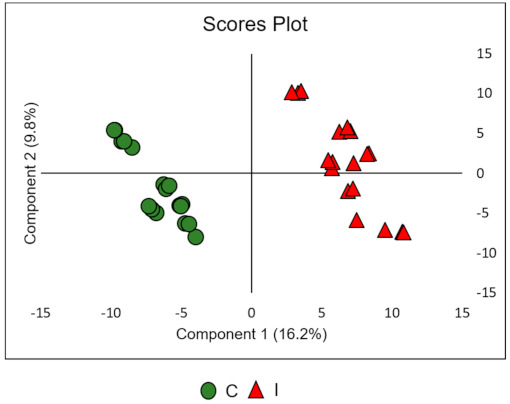
Partial least-squares discriminant analysis (PLS-DA) of 18 samples (triplicate of 6 control plants and 6 inoculated plants), green circles correspond to control plant samples (C), red circles correspond to inoculated plant samples (I). The classification model was built to screen potential biomarkers using VIP values.

The PLS-DA analysis (Figure 2), revealed the clear separation between the data set of the samples from control and inoculated plants by PLS-DA analysis. PLS-DA models obtained for the data set were evaluated using Leave one out cross-validation (LOOCV). The LOOCV consists of choosing one of the samples to compose the validation set and the other samples are used for the training set. A new sample is taken to compose the validation set and the sample that was used previously for the validation set this time will constitute the training set, these operations are repeated several times until all samples have been part of the validation set at least once [28].

Three parameters were used to validate the model, precision that describes how close to a true value measurement, *R*^2^ that measures the quality of the fit, and *Q^2^* that measures the predictive capacity of the model. The values obtained by cross-validation were: precision = 1.00, *R^2^* = 0.99 *Q^2^* = 0.96, these values represent a great performance for the model created with the data sets. VIP values ≥ 1 detected in the PLS-DA analysis associated with *p*-value data ≤ 0.001 (Welch *t*-test) were used for the selection of molecular features, which show a significant contribution to the observed clustering. Thirty-seven variables were selected as the most significant for the discrimination of the groups (Supplementary Table S1).

Despite the excellent values obtained by cross-validation demonstrating great precision, the model created cannot be used as a classification model for external samples because many of the variables used in the construction of this model have low intensities. These variables may be absent in external samples and lead to an incorrect classification. Thus, a new model using only the selected variables (Supplementary Table S1) with the most significant was created (Supplementary Figure S4A). The values obtained by cross-validation were: precision = 1.00, *R^2^* = 0.99 *Q^2^* = 0.99, presenting a performance similar to the previous model. The validation of the model was performed through permutation tests (Supplementary Figure S4B,C), with 2.000 repetitions for both tests. The probability that the model was created by chance was less than 0.0005%, showing a level of confidence that the separations are caused by the differences between the samples of control and inoculated plants. Thus, by reducing the number of non-significant variables in the model, it can overall be used as a classification strategy in further studies.

### 2.4. Chemical Classification and Identification of Metabolites by the GNPS Platform

The GNPS Molecular Networking platform was carried out to organize UHPLC-ESI (+)-MS/MS data and to obtain information on the molecular characteristics selected from the static and multivariate analysis through putative chemical classification (Supplementary Table S1). The MN tool correlates MS/MS spectra according to the similarity of fragmentation patterns of related precursor ions. In this way, the related compounds through their fragmentation profiles are grouped in clusters represented by a node, providing better visualization of the data and decreasing the data mining time of untargeted metabolomics studies. The analysis of the chemical map highlights structurally related compounds and, when comparing the MS/MS spectra with databases of the platform, facilitates the process of chemical classification and identification of the compounds.

The MolNetEnhancer analysis available on the GNPS platform was applied to the generated MN. MolNetEnhancer is a workflow that allows chemical annotation, visualization, and discovery of the subtle diversity of structures in molecular families [29]. The analysis combines complementary molecular mining tools with the MN network, such as the in silico annotation tool, network annotation propagation (NAP), and MS2LDA [30,31].

Once chemical compounds detected from the control plants and inoculated with *P. pachyrhizi* were combined, a total of 1.627 nodes (consensus spectra) were obtained in the MN (Supplementary Figure S5). Of these, 301 (18.5%) consensus spectra (brown nodes) were obtained exclusively when *P. pachyrhizi* was inoculated in soybean plants, 190 (11.8%) consensus spectra (green nodes) were obtained for plants in the control group, and 1.137 (69.8%) consensus spectra (gray nodes) obtained for both groups. Sixty-seven consensus spectra (black bold border nodes) were compatible with the GNPS library (4.18% of the total) which were manually confirmed with the MS/MS spectra (data not shown) through fragment ions and mass error, ranged from 0.0 to 6.7 ppm (Table 1).

**Figure 3:**
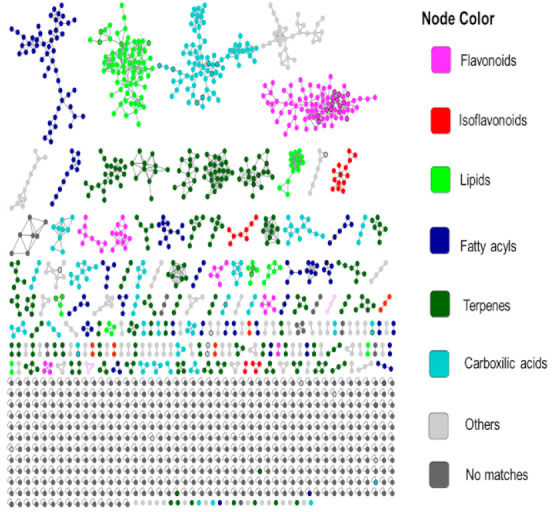
Molecular network of the MS/MS spectra obtained by the analysis of the soybean control plants, or inoculated with *P. pachyrhizi* colored by 6 chemical class terms selected as indicated in the legend annotated on the molecular network (Supplementary Figure S5) using the MolNetEnhancer. The black bold borders nodes represent the MS/MS spectra that had hits with the spectra of the GNPS libraries.

As shown in Figure 3, MolNetEnhancer provided the putative chemical classification of compounds detected from the control plants and inoculated with *P. pachyrhizi* and combined in the molecular network (Supplementary Figure S5), such as flavonoids, isoflavonoids, lipids, fatty acyls, terpenes, carboxylic acids, and others. The significantly regulated metabolites were assigned to the chemical classification to which they belong (Supplementary Table S1). Metabolites derived from phenylpropanoids, such as flavonoids and isoflavonoids, were up-regulated, while terpenes were down-regulated in inoculated plants compared to control plants.

**Figure 4:**
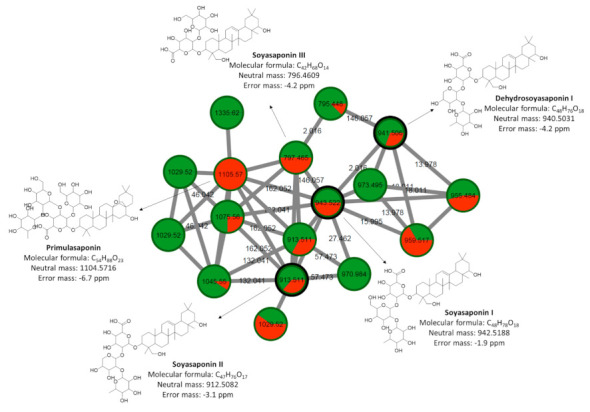
Cluster of terpenes containing triterpene saponins putatively characterized by molecular network obtained from MS/MS data from control plants and inoculated plants with *P. pachyrhizi*. The edge width represents the cosine score (0.88 to 0.99). The edge label represents the mass difference between nodes (2.016 Da, 15.995 Da, 18.011 Da, 132.041 Da, 146.058 Da, and 162.052 Da). The black bold borders nodes represent the MS/MS that had hits with the spectra of the GNPS libraries. The pie chart within each node corresponds to the percentage relative of the metabolite in the sample, green indicates soybean control plants, and red when soybean plants were inoculated with *P. pachyrhizi*.

The molecular network obtained provided a total of 191 clusters of interconnected nodes. The cluster represented in Figure 4, containing the spectra of MS/MS de [M + H]^+^ *m/z* 943.522, [M + H]^+^ *m/z* 941.506, [M + H]^+^ *m/z* 797.465, de [M + H]^+^ *m/z* 1105.57, de [M + H]^+^ *m/z* 913.511 putatively identified as soyasaponin I, dehydrosoyasaponin I, soyasaponin III, primulasaponin and soyasaponin II, respectively, highlight the potential of the molecular networking tool for identifying metabolites. These compounds are classified as terpenes, the analogs were separated by 2.016 Da, 15.995 Da, 18.011 Da, 132.041 Da, 146.058 Da, and 162.052 Da which are attributed to differences in H_2_, O, H_2_O, C_5_H_8_O_4,_ C_6_H_10_O_4_ e C_6_H_10_O_5_, respectively. All nodes showed greater spectral similarity, as they were grouped with relatively high cosine scores (0.88–0.99). Using this approach, other ions belonging to the same cluster could be putatively identified. A pie chart layout was also generated using the peak ion area in each sample group (control and inoculated plants) for qualitative assessment.

## 3. Discussion

Fatty acids and lipids play important roles in different stages of plant-pathogen interactions, including the supply of cellular energy to support metabolic processes, communication between the host and the pathogen, activation, and implementation of plant defense [32]. The lipoxygenase pathway, for example, provides the production of oxylipins, a wide range of metabolites generated by auto-oxidation or enzymatic oxidation of polyunsaturated fatty acids [33].

In our study, polyunsaturated fatty acids were putatively identified as linoleic acid [M + H]^+^ *m/z* 279.2318; 13-HPOT [M + H]^+^ *m/z* 311.2213; 12,13(S)-EOT [M + H]^+^ *m/z* 293.2108; 12-oxo-phytodienoic acid [M + H]^+^ *m/z* 293.2108, and OPC-8:0 [M + H]^+^ *m/z* 295.2266 (Table 1). These compounds are part of the biosynthesis pathway of the phytohormone jasmonic acid, also putatively identified in our study as [M + H]^+^ *m/z* 211.1331, the invasion by pathogens in the plant activates phospholipase enzymes in the plastid membrane, causing the synthesis of the linoleic acid precursor of the JA biosynthesis process [34]. Positive regulation of the signaling pathway mediated by jasmonic acid has already been reported in studies of resistance against ASR in *Medicago truncatula* [35] and *Arabidopsis thaliana* [36], non-host plants of *P. pachyrhizi*.

The amino acid phenylalanine is a precursor in the synthesis of phenylpropanoids, which is a group of secondary plant metabolites involved in plant defensive mechanisms against stressors [37,38,39]. The synthesis of phenylpropanoids is dependent on the enzyme phenylalanine ammonia-lyase (PAL), which catalyzes the conversion of phenylalanine to trans-cinnamic acid and ammonia. The trans-cinnamic acid can be incorporated into many different phenolic compounds, such as p-coumaric acid, caffeic acid, ferulic acid, and sinapic acid.

Transcriptomic studies provide evidence of the role of phenylpropanoid pathway genes in soybean resistance to ASR [40,41,42]. The increased resistance to specific isolates of *P. pachyrhizi* in soybean promoted by the *Rpp2* gene was compromised when genes, such as the GmPAL1, were silenced. Thus, phenylalanine and PAL might act on the resistance of soybean plants to ASR [43].

A wide range of phenolic compounds is derived from phenylpropanoid compounds such as flavonoids, isoflavonoids, and coumarins, which have been up-regulated in response to infection by *P. pachyrhizi* (Table 1). Flavonoids and isoflavonoids are phytoalexins, low molecular weight antimicrobial compounds, produced by plants in response to biotic stresses.

The aglycone daidzein [M + H]^+^ *m/z* 255.0650 and its malonyl daidzein conjugate [M + H]^+^ *m/z* 503.1174 (Table 1) are the main isoflavonoids observed in soybean grains and leaves. Daidzein is a precursor in the metabolic pathway that results in the production of soybean isoflavonoids [44]. In the study previously reported by us, using genotype susceptible to ASR, we observed through qualitative analysis a significant production of flavonoids and isoflavonoids in plants inoculated with *P. pachyrhizi*, as a plant defense response [24].

Coumarins have antimicrobial and antiviral activities and can act as important actors in the chemical defense strategy in plant–pathogen interactions [45]. Derivatives of the coumarin family have antioxidant and photosensitizing capabilities. Coumarins exposed to specific wavelengths can have nonspecific oxidative damage and cause subsequent death of fungal cells without affecting host cells [46,47]. In addition, the phytopathogenic fungus *Colletotrichum acutatum*, which causes citrus diseases, has been inactivated by the use of photo-treated coumarins and furocoumarins [48].

We identified four coumarins, 7-methoxycoumarin [M + H]^+^ *m/z* 177.0541; [M + H]^+^ *m/z* 193.0488 scopoletin; xanthyletin [M + H]^+^ *m/z* 229.0857, and osthole [M + H]^+^ *m/z* 245.1168 (Table 1). Beyer et al., 2019, demonstrated fungistatic activity of coumarin scopoletin against *P. pachyrhizi* [49]. Coumarin was able to suppress the formation of pre-infection streaks and penetration of *P. pachyrhizi* when sprayed on leaves of *Arabidopsis*, a non-host plant from ASR [49].

Terpenes are a broad class of compounds spread in plants, and play a role in defending plants against biotic and abiotic stress. Putatively identified triterpenic saponins (Table 1) are a group of phytoanticipins, preformed antimicrobial compounds, which act as chemical barriers against attack by pathogens [50]. Other studies have already reported the antifungal activity of saponins against plant pathogens [51,52]. These compounds have also been identified in the metabolome of the plant of genotype susceptible to ASR. However, the role of saponins against *P. pachyrhizi* is unknown.

## 4. Materials and Methods

### 4.1. Plant Preparation

Soybean (*Glycine max*) seeds of cultivar were used, PI567025A resistant to ASR (*Rpp3* gene) [25], provided by the Germplasm Active Bank (GAB) of Embrapa Soybean. The get seeds (daughters seeds) having homogeneous physiological characteristics were obtained from a single seed (mother seed). The experiment using the seeds was carried out in a greenhouse under the same growth conditions described in Silva et al. 2020 [24].

### 4.2. Preparation and Inoculation of the P. pachyrhizi

The spores of *P. pachyrhizi* were supplied by the Embrapa Soybean Phytopathology Laboratory (Londrina, Parana, Brazil), obtained from cultivar BRS 284 (susceptible standard), with 92% of germination viability. The inoculum was prepared as described in Silva et al. 2020 [24]. The soybean plants were divided into two groups: 6 control plants and 6 inoculated plants. Each plant was treated individually, by spraying with the fungal spore suspension and the control plants were treated by spraying (just excipients) with aid of a sprinkler bottle. After inoculation plants were submitted to automatic fogging (40 s) every two hours in greenhouse, for a period of 14 h, starting at dusk and kept under the same conditions (T = 28 ± 2 °C; UR = 70% ± 10% and photoperiod of 12 h/12 h (light: dark). Then the control plants were transferred to another greenhouse and kept under the same conditions to avoid contamination. Post-treatment harvesting of the plants was done for both cultivars at 14 days post-inoculation. by cutting off the leaves wrapped in aluminum foil, and immediately immersed in liquid nitrogen and transported to the laboratory. The samples were stored in an ultra-freezer (−80 °C) until the extraction time.

### 4.3. Metabolites Extraction

To evaluate the metabolic profile of the soybean leaves the metabolites were extracted from control plants and inoculated plants using a ternary solvent system (chloroform/methanol/water, 3:1:1 *v/v*). Soybean leaves were macerated, separately, using liquid nitrogen to preserve the sample during the process. The metabolite extraction was performed as described in Silva et al. 2020 [24]. The samples were concentrated in nitrogen flow and stored in a freezer at −20 °C until analysis.

### 4.4. UHPLC-ESI-MS/MS Analysis

The extracts were analyzed using ultra-high-performance liquid chromatography (Shimadzu, Nexera X2, Tokyo, Japan) coupled to a hybrid quadrupole time-of-flight high-resolution mass spectrometer (Impac II, Bruker Daltonics Corporation, Bremen, Germany) equipped with an electrospray ionization source. Chromatographic separation was performed with an Acquity UPLC HSS T3 C18 packed with 135 Å pore, 1.7 µm particle size, 2.1 × 100 mm column (Waters, Billerica, MA, USA) at a flow rate of 0.25 mL min^−1^. Elution gradient was carried out with a binary solvent system consisting of water with 0.1% formic acid (solvent A) and acetonitrile with 0.1% formic acid (solvent B). The initial conditions were 95% A and 5% B held for 1 min, the gradient was applied to 30% A and 70% B at 12 min, and changed to 2% A and 98% B at 20 min and maintained at 95% A and 5% B for 20 to 25 min at 40 °C, the final five minutes being intended for reconstitution of the column for the next analysis. The mass spectrometer was calibrated using a solution of sodium formate (10 mmol L^−1^; isopropanol:water; 1:1; v-v) containing 50 µL concentrated formic acid. The capillary voltage was operated in positive ionization modes set at 4500 V, with an endplate offset potential of −500 V. The dry gas parameters were set to 8 L min^−1^ at 180 °C with a nebulization gas pressure of 4 bar. Data were collected from *m/z* 50 to 1800 with an acquisition rate of 5 Hz, and the 5 ions of interest were selected by auto MS/MS scan fragmentation [24].

### 4.5. Data Preprocessing and Data Analysis

The raw data files from UHPLC-ESI(+)-MS/MS were exported as mzXML format and uploaded to the XCMS online software (https://xcmsonline.scripps.edu/, accessed date: 10 April 2020). The software was used for feature detection, retention time correction, alignment, automatic integration, and intensity. Parameter settings for XCMS data processing were as follows: Pairwise analysis was performed using centWave for feature detection (Δ*m*/*z* = 10 ppm, minimum peak width = 5 s, and maximum peak width = 20 s); for retention time correction and chromatograph alignment was performed with an obiwarp method (profStep = 1), minfrac = 0.5, bw = 5, mzwid = 0.015. Statistics analysis was performed with an unpaired parametric *t*-test (Welch *t*-test).

The processed data file (CSV format) was exported to MetaboAnalyst 3.0 (www.metaboanalyst.ca, accessed date: 10 April 2020) for the multivariate analysis. Prior features were normalized by sum and scaled by the pareto scaling method. Unsupervised multivariate methods Principal Component Analysis (PCA) was performed to determine differences in metabolic profiles between control and inoculated plants. The Partial Least Squares Discriminant Analysis (PLS-DA) supervised method was used to identify altered metabolites between groups.

The model built from PLS-DA analysis was validated using Leave-one-out cross-validation (LOOCV). The prediction capacity of the model was evaluated by the accuracy parameters R^2^ and Q^2^. Differentially expressed metabolites were selected according to the variable importance in projection (VIP) values ≥1 obtained from the PLS-DA model; *p*-value ≤ 0.001, from the Welch *t*-test and the maximum ion intensity ≥10,000.

### 4.6. Classical Molecular Networking Workflow Description

A molecular network was created using the online workflow (https://ccms-ucsd.github.io/GNPSDocumentation/) on the GNPS website (http://gnps.ucsd.edu, accessed date: 12 June 2020). The data were filtered by removing all MS/MS fragment ions within +/− 17 Da of the precursor *m*/*z*. MS/MS spectra were window filtered by choosing only the top 6 fragment ions in the +/− 50Da window throughout the spectrum. The precursor ion mass tolerance was set to 0.02 Da and an MS/MS fragment ion tolerance of 0.02 Da. A network was then created where edges were filtered to have a cosine score above 0.7 and more than 4 matched peaks. Further, edges between two nodes were kept in the network if and only if each of the nodes appeared in each other’s respective top 10 most similar nodes. Finally, the maximum size of a molecular family was set to 100, and the lowest-scoring edges were removed from molecular families until the molecular family size was below this threshold. The spectra in the network were then searched against GNPS’ spectral libraries. The library spectra were filtered in the same manner as the input data. All matches kept between network spectra and library spectra were required to have a score above 0.7 and at least 4 matched peaks.

### 4.7. MolNetEnhancer Workflow Description for Chemical Class Annotation of Molecular Networks

To enhance chemical structural information within the molecular network, information from in silico structure annotations from GNPS Library Search, Network Annotation Propagation were incorporated into the network using the GNPS MolNetEnhancer workflow (https://ccms-ucsd.github.io/GNPSDocumentation/molnetenhancer/) on the GNPS website (http://gnps.ucsd.edu, accessed date: 12 June 2020). Chemical class annotations were performed using the ClassyFire chemical ontology.

## 5. Conclusions

*Phakopsora pachyrhizi* is one of the most challenging pathogens that attack soybean crops, its high genetic variability makes it difficult to control ASR disease, requiring extensive and continuous research to control and combat it. The application of the untargeted metabolomics approach using mass spectrometry combined with chemometric analysis and Global Natural Products Social Molecular Networking platform in the interaction soybean–*Phakopsora pachyrhizi* was efficient for the knowledge of the expression of metabolites associated with soybean genotype interaction under study and can be used in other plant genotypes soybean, as well as other plant–pathogen interactions. The metabolome of the resistant genotype plant presented secondary metabolites similar to the metabolome of the susceptible genotype plant, showing the same pathways related to the plant’s defense responses, such as phenylpropanoids, flavonoids, and isoflavonoids. Besides, this metabolomic study corroborates the transcriptomic studies of ASR already carried out, which report the induction of phenylpropanoid, flavonoid, and isoflavonoid metabolic pathway genes as a defense response to *P. pachyrhizi*, which could assist in research on the development of plant genes more resistant to *P. pachyrhizi* and also in the development of more effective and specific products against the disease.

## Figures and Tables

**Table 1:** Metabolites are putatively identified in soybean plants.

Putative Metabolite Identification	Molecular Formula	[M + H]^+^ Measured	[M + H]^+^ Theoretical	Mass Accuracy (ppm)
Amino acid				
Proline	C_5_H_9_NO_2_	116.0704	116.0706	−1.7
Pipecolic acid	C_6_H_11_NO_2_	130.0861	130.0863	−1.5
Leucine	C_6_H_13_NO_2_	132.1020	132.1019	0.8
Phenylalanine	C_9_H_11_NO_2_	166.0859	166.0863	−2.4
Tyrosine	C_9_H_11_NO_3_	182.0807	182.0812	−2.7
Tryptophan	C_11_H_12_N_2_O_2_	205.0964	205.0972	−3.9
Phenylpropanoids				
p-Coumaric acid	C_9_H_8_O_3_	165.0541	165.0546	−3.0
Citric acid	C_6_H_8_O_7_	193.0337	193.0343	−3.1
Ferulic acid	C_10_H_10_O_4_	195.0647	195.0642	2.6
Abscisic acid	C_15_H_20_O_4_	265.1425	265.1434	−3.4
Peptides				
Ile-Pro	C_11_H_20_N_2_O_3_	229.1542	229.1546	−1.7
Ile-Val	C_11_H_22_N_2_O_3_	231.1700	231.1703	−1.3
Leu-Leu	C_12_H_24_N_2_O_3_	245.1855	245.1859	−1.6
Leu-Asn	C_10_H_19_N_3_O_4_	246.1439	246.1448	−3.7
Asp-Leu	C_10_H_18_N_2_O_5_	247.1279	247.1288	−3.6
Pro-Phe	C_15_H_22_N_2_O_3_	263.1412	263.1390	8.4
Phe-Val	C_14_H_20_N_2_O_3_	265.1543	265.1546	−1.1
Leu-Phe	C_14_H_18_N_2_O_3_	279.1694	279.1703	−3.2
Asn-Phe	C_13_H_17_N_3_O_4_	280.1292	280.1291	0.4
Leu-Leu-Gly	C_14_H_27_N_3_O_4_	302.2067	302.2074	−2.3
Peptide	C_15_H_27_N_3_O_4_	314.2066	314.2074	−2.5
Leu-Val-Val	C_16_H_31_N_3_O_4_	330.2377	330.2387	−3.0
Leu-Leu-Val	C_17_H_33_N_3_O_4_	344.2533	344.2543	−2.9
Cumarin				
7-Methoxycoumarin	C_10_H_8_O_3_	177.0541	177.0546	−2.8
Scopoletin	C_10_H_8_O_4_	193.0488	193.0495	−3.6
Xanthyletin	C_14_H_12_O_3_	229.0857	229.0859	−0.9
Osthole	C_15_H_16_O_3_	245.1168	245.1172	−1.6
Flavonoids				
Daidzin	C_21_H_20_O_9_	417.1195	417.1180	3.6
Daidzein	C_15_H_10_O_4_	255.0650	255.0651	−0.4
Neobavaisoflavone	C_20_H_18_O_4_	323.1273	323.1278	−1.5
Sojagol	C_20_H_16_O_5_	337.1067	337.1070	−0.9
Isoflavonoid	C_21_H_20_O_4_	337.1430	337.1434	−1.2
Gliceollin I	C_20_H_18_O_5_	339.1217	339.1227	−2.9
Gliceollin II	C_20_H_18_O_5_	339.1222	339.1227	−1.5
Gliceollin III	C_20_H_18_O_5_	339.1220	339.1227	−2.1
Isoflavonoid	C_21_H_18_O_5_	351.1219	351.1227	−2.3
Isoflavonoid	C_21_H_20_O_5_	353.1377	353.1383	−1.7
Xanthohumol	C_21_H_22_O_5_	355.1534	355.1540	−1.7
7-O-Methylluteone	C_21_H_20_O_6_	369.1330	369.1333	−0.7
Schizandrin C	C_22_H_24_O_6_	385.1638	385.1645	−1.8
Genistin	C_21_H_20_O_10_	433.1118	433.1129	−2.5
Luteolin 8-C-glucoside	C_21_H_21_O_11_	449.1067	449.1078	−2.4
Isoquercitin	C_21_H_20_O_12_	465.1020	465.1027	−1.5
3’-O-methyltricetin 3-O-α-L-rhamnopyranoside	C_22_H_22_O_12_	479.1170	479.1184	−2.9
Kaempferol-O-acetylhexoside	C_23_H_22_O_12_	491.1163	491.1184	−4.3
Malonyldaidzin	C_24_H_22_O_12_	503.1174	503.1184	−2.0
Formononetin 7-O-glucoside-6’’’’-O-malonate	C_25_H_24_O_12_	517.1339	517.1341	−0.3
Malonylgenistin	C_24_H_22_O_13_	519.1129	519.1133	−0.8
Isoorientin 2’’’’-O-rhamnoside	C_27_H_30_O_15_	595.1652	595.1657	−0.8
Rutin	C_27_H_30_O_16_	611.1588	611.1607	−3.0
Narcissin	C_28_H_32_O_16_	625.1747	625.1763	−2.6
Robinin	C_33_H_40_O_19_	741.2209	741.2236	−3.6
Flavonoid-7-O-glycosides	C_33_H_40_O_20_	757.2168	757.2186	−2.3
Flavonoid-7-O-glycosides	C_34_H_42_O_20_	771.2316	771.2342	−3.4
Lipids				
Jasmonic acid	C_12_H_18_O_3_	211.1331	211.1328	1.4
(9Z,12Z,15Z)-octadeca-9,12,15-trien-6-ynoic acid	C_18_H_26_O_2_	275.2003	275.2005	−0.7
13S-Hydroxy-9Z,11E,15Z-octadecatrienoic acid	C_18_H_28_O_2_	277.2153	277.2162	−3.2
Linolenic acid	C_18_H_31_O_2_	279.2318	279.2318	0.0
15-Methylhexadecasphinganine	C_17_H_37_NO_2_	288.2890	288.2897	−2.4
12,13(S)-EOT	C_18_H_28_O_3_	293.2108	293.2111	−1.0
12-OPDA	C_18_H_28_O_3_	293.2108	293.2111	−1.0
10,13-Nonadecadiynoic acid	C_19_H_30_O_2_	291.2320	291.2318	0.7
OPC-8:0	C_18_H_30_O_3_	295.2266	295.2267	−0.3
13-HPOT	C_18_H_30_O_4_	311.2213	311.2216	−1.0
Terpenes				
Soyasaponin III	C_42_H_68_O_14_	797.4641	797.4681	−5.0
Soyasaponin II	C_47_H_76_O_17_	913.5127	913.5155	−3.1
Dehydrosoyasaponin I	C_48_H_76_O_18_	941.5064	941.5104	−4.2
Soyasaponin I	C_48_H_78_O_18_	943.5243	943.5261	−1.9
Saponin	C_55_H_70_O_14_	955.4855	955.4838	1.8
Asiaticoside	C_48_H_78_O_19_	959.5182	959.5210	−2.9
Cauloside D	C_53_H_86_O_22_	1075.5636	1075.5683	−4.4
Jujuboside B	C_52_H_84_O_21_	1045.5540	1045.5547	−0.7
Primulasaponin	C_53_H_86_O_22_	1105.5715	1105.5789	−6.7

## Data Availability

The data presented in this study are available in article.

## Supplementary Materials

The following are available online at https://www.mdpi.com/2218-1989/11/3/179/s1, Figure S1: ASR symptoms, dark spots on the leaf, Figure S2: Reddish-brown lesion type of ASR, Figure S3: Base peak chromatogram (BPC) of analytical replicates after UHPLC-ESI(+)-MS/MS analysis. (**A**) Triplicate plant 1; (**B**) Triplicate plant 2; (**C**) Triplicate plant 3; (**D**) Triplicate plant 4; (**E**) Triplicate plant 5; (**F**) Triplicate plant 6. Green BPCs: control plants, red BPCs: inoculated plants, Figure S4: (**A**) PLS-DA of 18 samples (triplicate of 6 control plants and 6 inoculate plants), green circles correspond to control plant samples (C), red circles correspond to inoculated plant samples (I). The model was built using potential biomarkers obtained by the VIP values. (**B**) PLS-DA permutation validation evaluated by group separation distance (permutation number = 2000). (**C**) PLS-DA permutation validation by prediction accuracy (permutation number = 2000), Figure S5: Molecular Network of the MS/MS spectra obtained by the analysis of the soybean control plants, or inoculated with *P. pachyrhizi*. Green nodes correspond to the MS/MS spectra of soybean control plants. Red nodes correspond to the MS/MS spectra of soybean plants inoculated with *P. pachyrhizi*. Pink nodes correspond to the MS/MS spectra shared spectra in both samples. The edge width represents the cosine score (0.7 to 1.0). The black bold borders nodes represent the MS/MS spectra that had hits with the spectra of the GNPS libraries, Table S1: Metabolites significantly regulated in soy in response to infection by *P. pachyrhizi* and putative chemical classification.
