# Supplementary material for: Untargeted Metabolomics Analysis by UHPLC-MS/MS of Soybean Plant in a Compatible Response to Phakopsora pachyrhizi Infection
Source: Metabolites. 2021 Mar 19;11(3):179. doi: 10.3390/metabo11030179 (PMC8003322; doi:10.3390/metabo11030179)
Supplement: Supplementary file 1 [file metabolites-11-00179-s001.pdf]

Supplementary Material

Untargeted metabolomics analysis by UHPLC-MS/MS of soybean plant in a compatible response to *Phakopsora pachyrhizi* infection.

Evandro Silva[1], José Perez da Graça[2], Carla Porto[1], Rodolpho Martin do Prado[1], Estela Nunes[3], Francismar Correa Marcelino Guimaraes[2], Mauricio Conrado Meyer[2] and Eduardo Jorge Pilau[1]*

[1] Laboratory of Biomolecules and Mass Spectrometry, Department of Chemistry, State University of Maringá, 5790, Colombo Av, CP 87020-080, Maringá, PR, Brazil.

[2] Brazilian Agricultural Research Corporation Soybean, Carlos João Strass Rd, Londrina, PR, 86001-970, Brazil.

[3] Brazilian Agricultural Research Corporation Swine & Poultry, BR-153, Km 110 Rd, CP 89715-899, Concórdia, SC, Brazil.

*Correspondence
Dr. Eduardo Jorge Pilau
e-mail address: ejpilau@uem.br
Telephone number: +55 44 3011-5098

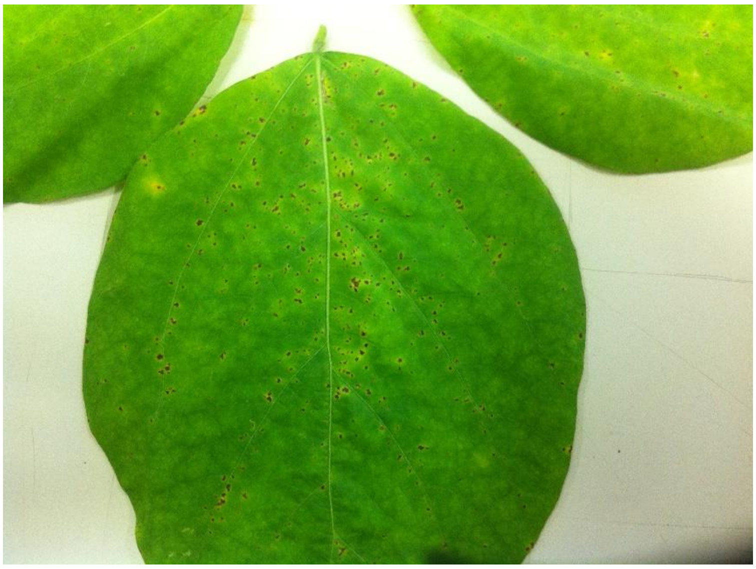

Figure S1. ASR symptoms, dark spots on the leaf.

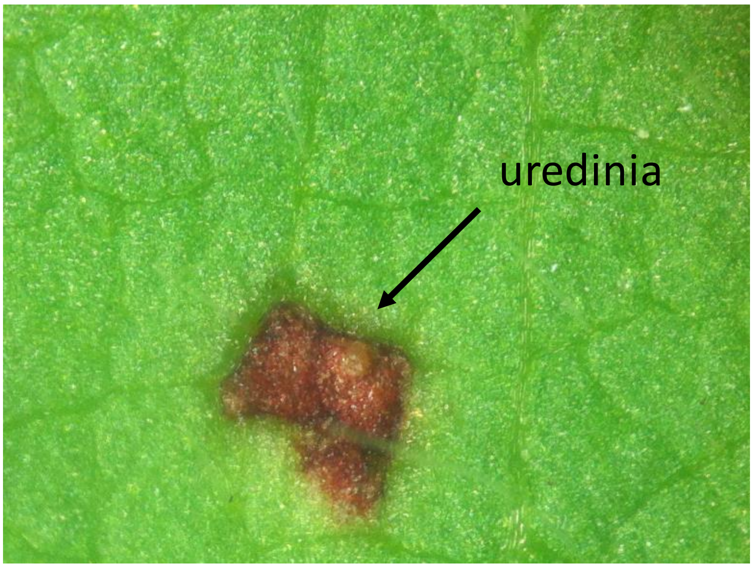

Figure S2. Reddish-brown lesion type of ASR.

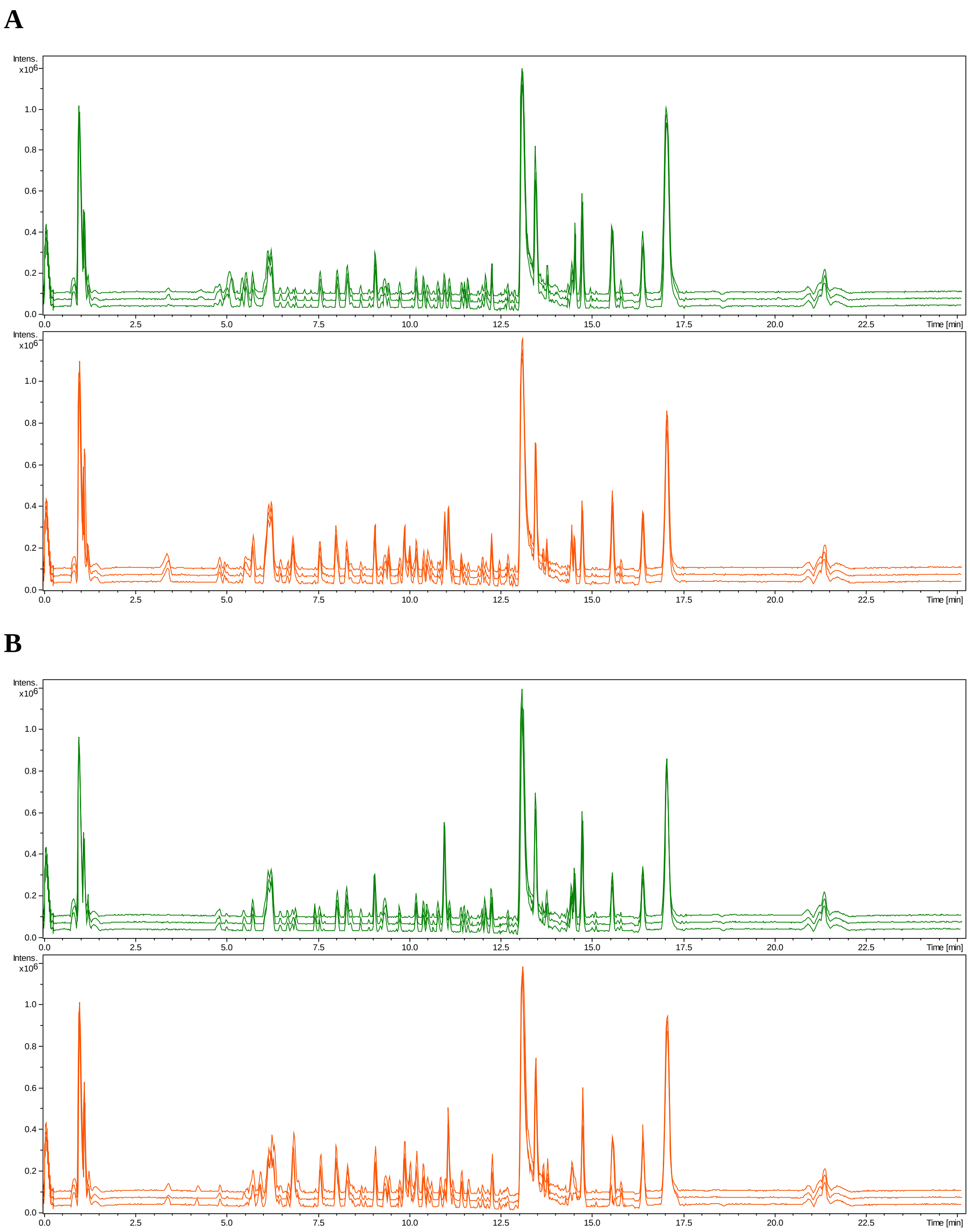

A
Intens.
x10^6
1.0
0.8
0.6
0.4
0.2
0.0
0.0
2.5
5.0
7.5
10.0
12.5
15.0
17.5
20.0
22.5
Time [min]
B

C

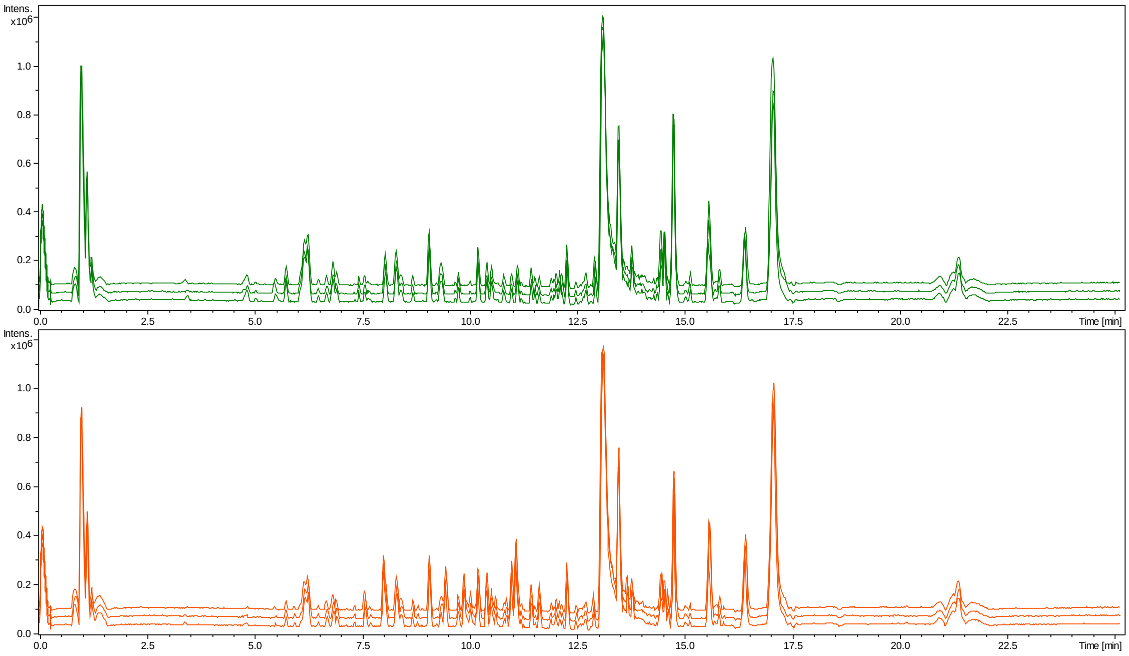
Intens.
x10^6
1.0
0.8
0.6
0.4
0.2
0.0
0.0
2.5
5.0
7.5
10.0
12.5
15.0
17.5
20.0
22.5
Time [min]

D

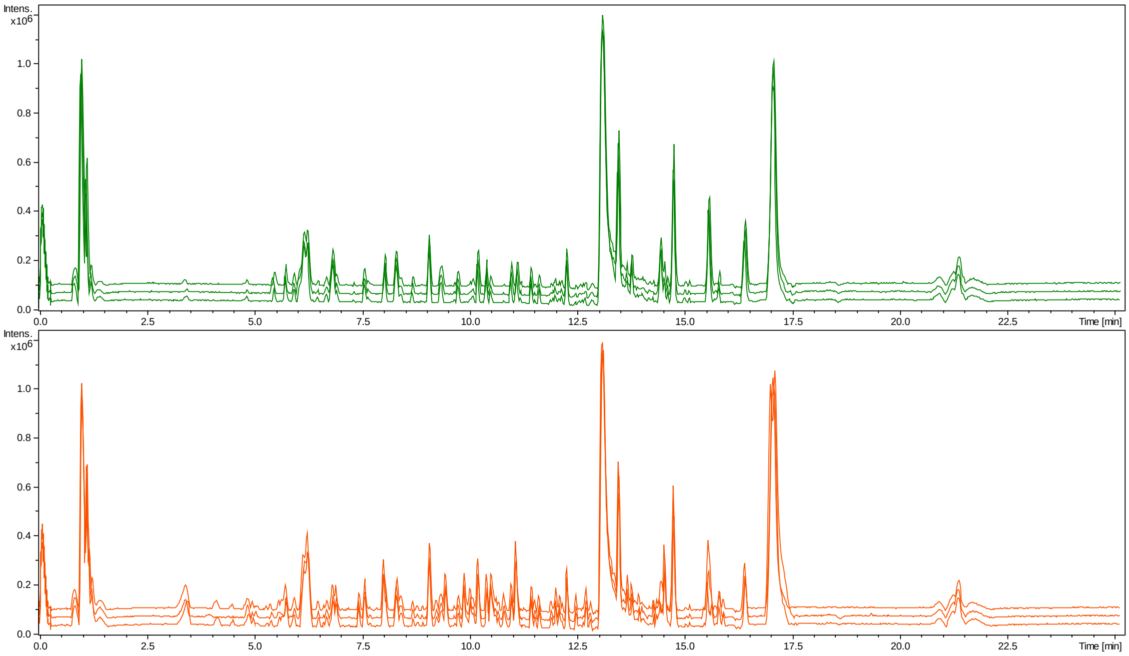
Intens.
x10^6
1.0
0.8
0.6
0.4
0.2
0.0
0.0
2.5
5.0
7.5
10.0
12.5
15.0
17.5
20.0
22.5
Time [min]

E

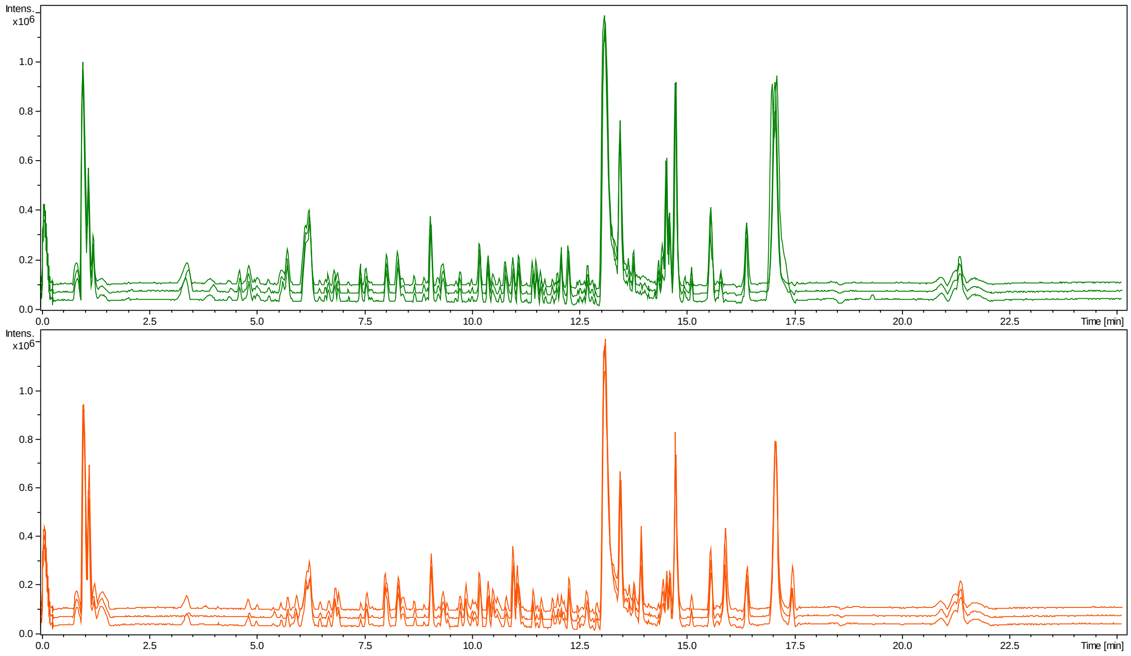

F

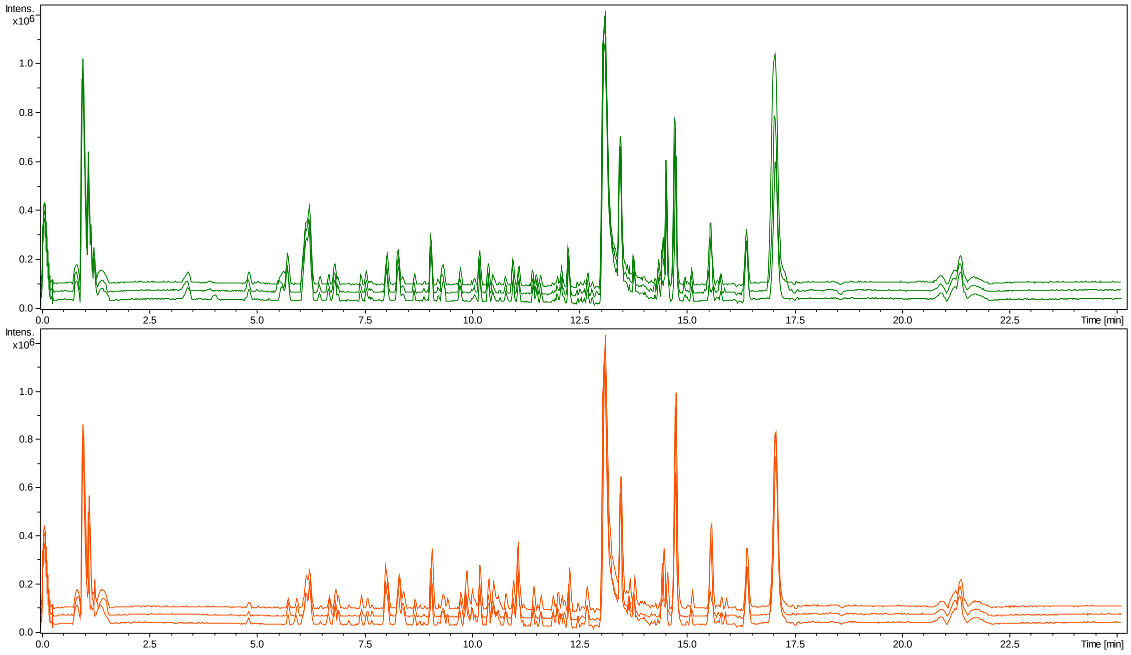

Figure S3. Base peak chromatogram (BPC) of analytical replicates after UHPLC-ESI(+)-MS/MS analysis. A) Triplicate plant 1; B) Triplicate plant 2; C) Triplicate plant 3; D) Triplicate plant 4; E) Triplicate plant 5; F) Triplicate plant 6. Green BPCs: control plants, red BPCs: inoculated plants.

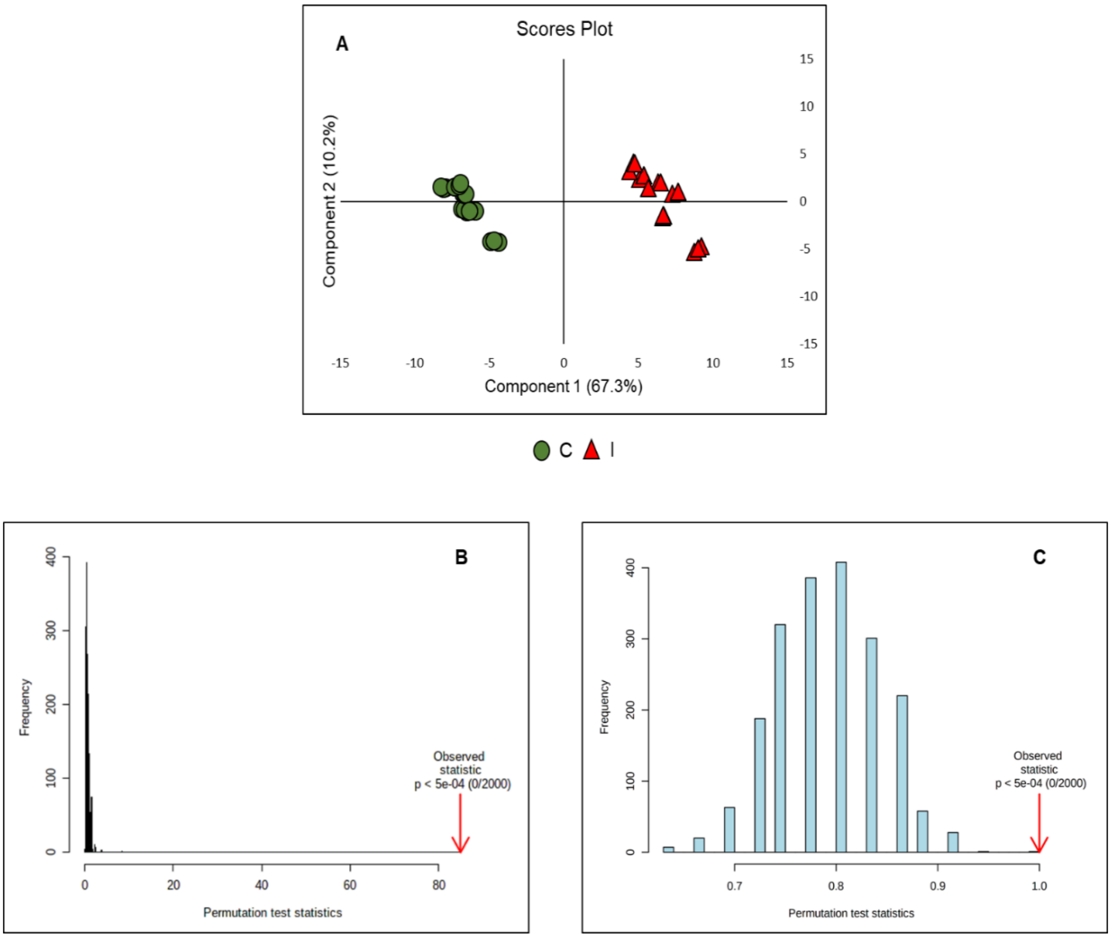

Figure S4. (A) PLS-DA of 18 samples (triplicate of 6 control plants and 6 inoculated plants), green circles correspond to control plant samples (C), red circles correspond to inoculated plant samples (I). The model was built using potential biomarkers obtained by the VIP values. **(B)** PLS-DA permutation validation evaluated by group separation distance (permutation number = 2000). **(C)** PLS-DA permutation validation by prediction accuracy (permutation number = 2000).

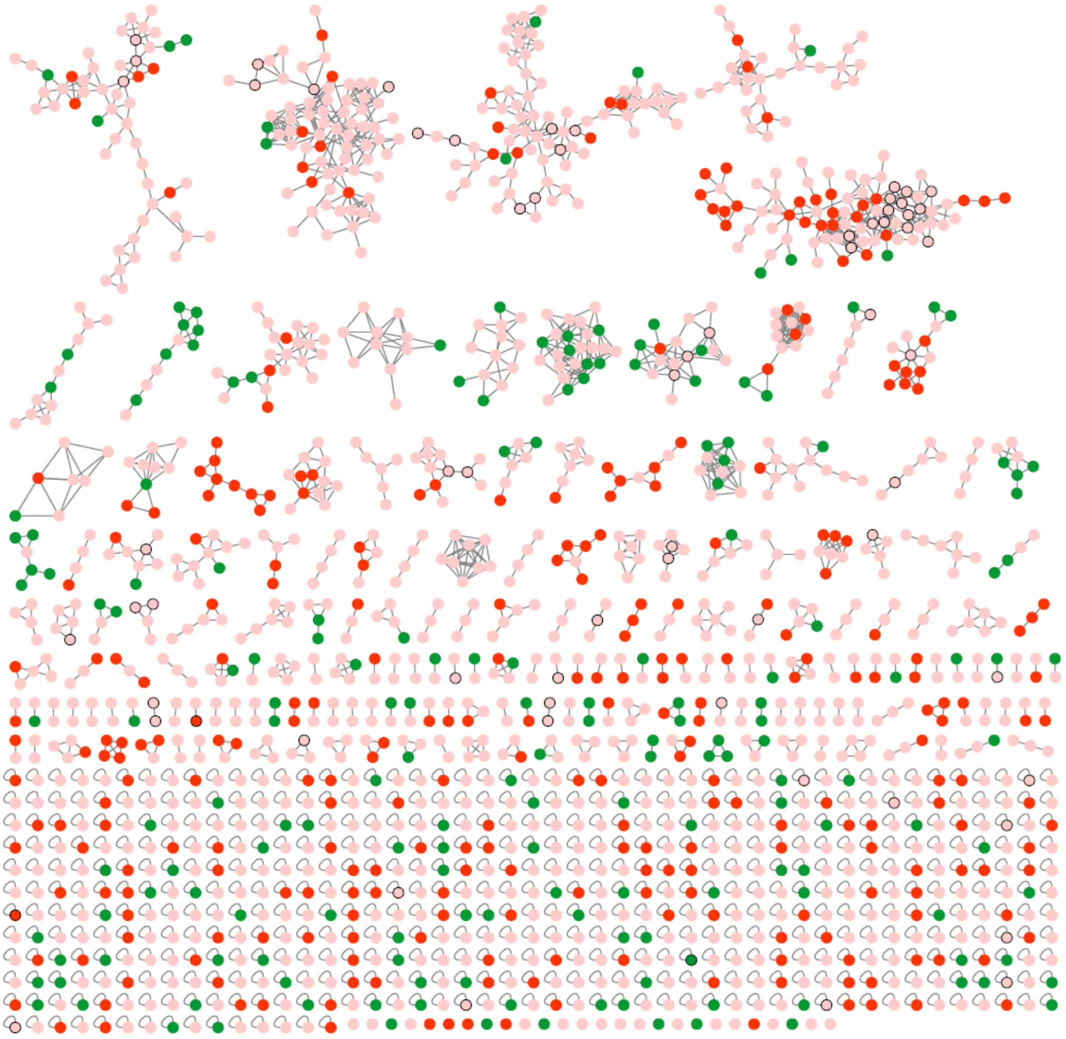

Figure S5. Molecular Network of the MS/MS spectra obtained by the analysis of the soybean control plants, or inoculated with *P. pachyrhizi*. Green nodes correspond to the MS/MS spectra of soybean control plants. Red nodes correspond to the MS/MS spectra of soybean plants inoculated with *P. pachyrhizi*. Pink nodes correspond to the MS/MS spectra shared spectra in both samples. The edge width represents the cosine score (0.7 to 1.0). The black bold borders nodes represent the MS/MS spectra that had hits with the spectra of the GNPS libraries.

Table S1: Metabolites significantly regulated in soy in response to infection by *P. pachyrhizi* and putative chemical classification.

ID	RT (min)	m/z	VIP	p-value	regulation	Chemical class	Molecular Family
1	4.67	188.0702	2.8	8.66E-03	DOWN	Amino acid	Organic acids and derivatives
2	6.47	933.2624	1.7	1.19E-04	DOWN	Carboxylic acid	Organic acids and derivatives
3	9.91	321.1118	10.9	4.60E-11	UP	Coumarin	Phenylpropanoids
4	10.18	259.0954	4.2	2.29E-09	UP	Coumarin	Phenylpropanoids
5	9.91	229.0855	3.2	3.11E-14	UP	Coumarin	Phenylpropanoids
6	10.80	321.1119	2.6	1.92E-13	UP	Coumarin	Phenylpropanoids
7	10.54	333.2030	4.3	5.16E-05	UP	Fatty Acyls	Lipids and lipid-like molecules
8	11.54	291.1948	3.8	3.90E-04	DOWN	Fatty Acyls	Lipids and lipid-like molecules
9	12.50	279.2313	2.6	1.01E-04	UP	Fatty Acyls	Lipids and lipid-like molecules
10	5.64	345.1444	2.5	2.36E-04	UP	Fatty Acyls	Lipids and lipid-like molecules
11	6.33	503.1176	5.2	3.74E-03	UP	Flavonoid glycosides	Phenylpropanoids
12	5.86	611.1592	4.2	7.47E-04	UP	Flavonoid glycosides	Phenylpropanoids
13	6.14	465.1026	4.1	2.38E-04	UP	Flavonoid glycosides	Phenylpropanoids
14	6.37	533.1280	3.9	3.65E-03	UP	Flavonoid glycosides	Phenylpropanoids
15	9.91	339.1223	3.5	8.51E-13	UP	Flavonoid glycosides	Phenylpropanoids
16	7.61	547.1433	3.2	7.35E-03	UP	Flavonoid glycosides	Phenylpropanoids
17	6.35	503.1177	2.6	3.74E-03	UP	Flavonoid glycosides	Phenylpropanoids
18	14.11	731.4311	2.4	5.67E-09	UP	Glycerophospholipid	Lipids and lipid-like molecules
19	11.11	337.1430	13.0	1.18E-11	UP	Isoflavonoid	Phenylpropanoids
20	11.76	337.1067	11.8	1.11E-15	UP	Isoflavonoid	Phenylpropanoids
21	10.18	351.1219	5.4	2.89E-09	UP	Isoflavonoid	Phenylpropanoids
22	11.23	367.1535	5.0	5.85E-08	UP	Isoflavonoid	Phenylpropanoids
23	10.18	369.1330	4.8	2.26E-09	UP	Isoflavonoid	Phenylpropanoids
24	10.26	323.1273	4.0	6.28E-07	UP	Isoflavonoid	Phenylpropanoids
25	9.41	323.1272	3.3	3.63E-11	UP	Isoflavonoid	Phenylpropanoids

26	11.09	355.1534	3.2	1.36E-11	UP	Isoflavonoid	Phenylpropanoids
27	11.23	385.1638	2.7	1.64E-07	UP	Isoflavonoid	Phenylpropanoids
28	10.18	351.1221	2.5	2.89E-09	UP	Isoflavonoid	Phenylpropanoids
29	8.40	353.1377	3.5	1.61E-05	UP	Linear 1,3-diarylpropanoids	Phenylpropanoids
30	6.35	250.1442	2.6	1.38E-05	DOWN	Organooxygen compound	Organic oxygen compounds
31	13.12	579.2916	7.3	2.54E-03	DOWN	Sesquiterpenoid	Terpene
32	18.07	661.4627	4.8	7.54E-11	DOWN	Triterpenoid	Terpene
33	9.37	943.5243	4.5	4.57E-06	DOWN	Triterpene saponins	Terpene
34	12.12	331.1871	4.4	4.97E-04	DOWN	Terpene	Terpene
35	15.99	507.3640	3.8	2.27E-06	DOWN	Prenol lipids	Terpene
36	13.70	502.3152	2.5	2.31E-08	UP	Prenol lipids	Terpene
37	9.97	207.1376	1.9	2.53E-03	DOWN	Prenol lipids	Terpene
